# Mobile Apps for Hematological Conditions: Review and Content Analysis Using the Mobile App Rating Scale

**DOI:** 10.2196/32826

**Published:** 2022-02-16

**Authors:** Álvaro Narrillos-Moraza, Patricia Gómez-Martínez-Sagrera, Miguel Ángel Amor-García, Vicente Escudero-Vilaplana, Roberto Collado-Borrell, Cristina Villanueva-Bueno, Ignacio Gómez-Centurión, Ana Herranz-Alonso, María Sanjurjo-Sáez

**Affiliations:** 1 Servicio de Farmacia, Hospital General Universitario Gregorio Marañón Madrid Spain; 2 Servicio de Hematología, Hospital General Universitario Gregorio Marañón Madrid Spain

**Keywords:** blood, hematology, mHealth, mobile apps, quality, rating tool, mobile phone

## Abstract

**Background:**

Hematological conditions are prevalent disorders that are associated with significant comorbidities and have a major impact on patient care. Concerning new tools for the care of these patients, the number of health apps aimed at hematological patients is growing. Currently, there are no quality analyses or classifications of apps for patients diagnosed with hematological conditions.

**Objective:**

The aim of this study is to analyze the characteristics and quality of apps designed for patients diagnosed with hematological conditions by using the Mobile App Rating Scale (MARS).

**Methods:**

We performed an observational, cross-sectional descriptive study of all smartphone apps for patients diagnosed with hematological conditions. A search was conducted in March 2021 using the following terms: *anemia*, *blood cancer*, *blood disorder*, *hematological cancer*, *hematological malignancy*, *hematological tumor*, *hematology*, *hemophilia*, *hemorrhage*, *lymphoma*, *leukemia*, *multiple myeloma*, *thalassemia*, *thrombocytopenia*, and *thrombosis*. The apps identified were downloaded and evaluated by 2 independent researchers. General characteristics were registered, and quality was analyzed using MARS scores. Interrater reliability was measured by using the Cohen κ coefficient.

**Results:**

We identified 2100 apps in the initial search, and 4.19% (88/2100) of apps met the inclusion criteria and were analyzed. Of the 88 apps, 61% (54/88) were available on Android, 30% (26/88) were available on iOS, and 9% (8/88) were available on both platforms. Moreover, 7% (6/88) required payment, and 49% (43/88) were updated in the last year. Only 26% (23/88) of the apps were developed with the participation of health professionals. Most apps were informative (60/88, 68%), followed by preventive (23/88, 26%) and diagnostic (5/88, 6%). Most of the apps were intended for patients with anemia (23/88, 26%). The mean MARS score for the overall quality of the 88 apps was 3.03 (SD 1.14), ranging from 1.19 (lowest-rated app) to 4.86 (highest-rated app). Only 47% (41/88) of the apps obtained a MARS score of over 3 points (acceptable quality). Functionality was the best-rated section, followed by aesthetics, engagement, information, and app subjective quality. The five apps with the highest MARS score were the following: *Multiple Myeloma Manager*, *Hodgkin Lymphoma Manager*, *Focus On Lymphoma*, *ALL Manager*, and *CLL Manager*. The analysis by operating system, developer, and cost revealed statistically significant differences in MARS scores (*P*<.001, *P*<.001, and *P*=.049, respectively). The interrater agreement between the 2 reviewers was substantial (k=0.78).

**Conclusions:**

There is great heterogeneity in the quality of apps for patients with hematological conditions. More than half of the apps do not meet acceptable criteria for quality and content. Most of them only provide information about the pathology, lacking interactivity and personalization options. The participation of health professionals in the development of these apps is low, although it is narrowly related to better quality.

## Introduction

### Background

The use of mobile technologies for health is increasing at an unstoppable rate. App capabilities for sharing health care information or real-time patient monitoring make them an important health tool because of their ease of use, broad reach, and wide acceptance [1]. At the beginning of 2021, more than 53,000 medical apps were available in the Android Play Store (one of the main download platforms) [2]. Medical apps have targeted a diverse number of conditions, such as diabetes [3,4], pain [5], rheumatic [6] and psychiatric disorders [7], COVID-19 [8-10], or cancer [11-13]. Apps for patients diagnosed with hematological conditions are also found on the main download platforms, although there is little information about them.

Hematological conditions comprise a wide range of disorders that can be classified as nonmalignant (anemia, hemorrhagic, or thrombotic disorders and conditions affecting blood-forming organs) and malignant (hematological cancers, such as Hodgkin and non-Hodgkin lymphoma, leukemia, or multiple myeloma, among others) [14]. These diseases meet all criteria for qualifying as a very important public health problem, with serious morbidities affecting patients worldwide [14-16]. Many of these conditions, such as hemophilia or anemia, are highly prevalent and become chronic. These patients could benefit from tools that improve treatment adherence or self-management guidelines, making medical apps an increasingly attractive option for this purpose [17,18].

Considering the large number of health apps available for patients with hematological conditions and the increasing interest in tools that encourage patient self-care, a proper review is needed. However, no clear consensus exists as to the appropriate method to assess the quality of health apps [19]. The Mobile App Rating Scale (MARS) is the most widely used scale for evaluating the quality and content of health apps. This allows the evaluation and comparison of apps by relating to their user engagement, functionality, aesthetics, and information quality [20,21]. In addition, it provides a quantitative and validated system that allows both users and health care professionals to avoid unreliable information.

### Objective

The aim of our study is to analyze the characteristics and quality of mobile apps for patients diagnosed with hematological conditions using the MARS.

## Methods

### Study Design

We performed an observational, descriptive, cross-sectional study of all smartphone apps for patients diagnosed with hematological conditions, including hematological malignancies, various types of anemia, and hemorrhagic and thrombotic diathesis, available on the Android and iOS platforms. The study followed the PRISMA-P (Preferred Reporting Items for Systematic Review and Meta-Analysis Protocols) 2015 guidelines for systematic reviews [22].

### Eligibility Criteria

A search on the Apple App Store and Android Play Store was performed in February 2021 by 2 independent health professionals with experience in app analysis, design, and development (PGMS and ANM). The following search terms were used: *anemia*, *blood cancer*, *blood disorder*, *hematological cancer*, *hematological malignancy*, *hematological tumor*, *hematology*, *hemophilia*, *hemorrhage*, *lymphoma*, *leukemia*, *multiple myeloma*, *thalassemia*, *thrombocytopenia*, and *thrombosis*. The reviewers screened the title and download page of the apps. Only apps intended for patients or their caregivers and in English or Spanish were selected. Those apps potentially eligible were downloaded and installed on the appropriate, corresponding mobile device, regardless of the cost. iOS apps were installed on an iPhone 7 (version 14.4.2; Apple Inc) and Android apps on a Nexus 5X (Android version 8.1.0; Google LLC). Apps with nonscientific content; intended for health care professionals; duplicated; not specific for hematological conditions; specific to congresses, meetings, and charitable purposes; and those with restricted access were excluded from the review.

### Data Extraction and Quality Assessment

Apps were individually evaluated in isolation by the same 2 independent reviewers. Variables analyzed were app name, search term (for what the app was found), platform (Android or iOS), developer, hematological disorder, cost, app category (books and reference works, education, entertainment, health and fitness, health and wellness, lifestyle, medicine, simulation, and social media), date of the last update, language, and purpose. Concerning the developer, if hospitals, health authorities, universities, scientific societies, or patients’ associations were involved in the design of an app, we classified them as *developed by a health organization*. The purpose was further classified into the following categories: diagnostic, informative, and preventive depending on whether the priority of the app was to run self-diagnosis, to provide generic data about one or several conditions, or to track treatment and symptoms, respectively. Grading was assessed by the same 2 independent reviewers according to the validated MARS. Data extraction, analysis, and grading were completed within 60 days.

The MARS is a multidimensional instrument that assesses the quality of mobile health apps. The quality assessment consists of a total of 23 items covering 5 dimensions. The dimensions are (1) engagement (5 items: entertainment, interest, customization, interactivity, and target adequacy), (2) functionality (4 items: performance, ease of use, navigation, and gestural design), (3) aesthetics (3 items: layout, graphics, and visual appeal), (4) information quality (7 items: accuracy of app description, goals, quality of information, quantity of information, quality of visual information, evidence base, and credibility), and (5) subjective quality (4 items: recommendation, payment willingness, frequency of use, and overall rating). All items were rated on a 5-point scale (1=inadequate; 2=poor; 3=acceptable; 4=good; 5=excellent). Then, the overall quality of the app was obtained from the mean score of the domains [20,21].

### Data Analysis

Quantitative variables were expressed as means and SDs and categorical variables as frequencies and percentages. Continuous variables were compared using the 2-tailed *t* test when the distribution was normal or the Mann-Whitney test when it was not. κ coefficient was used to measure the interrater reliability of the data analyzed by the 2 independent researchers [23]. Data were analyzed using Stata (version IC-16; StataCorp). A *P* value <.05 was considered statistically significant.

## Results

### Overview

**Figure 1:**
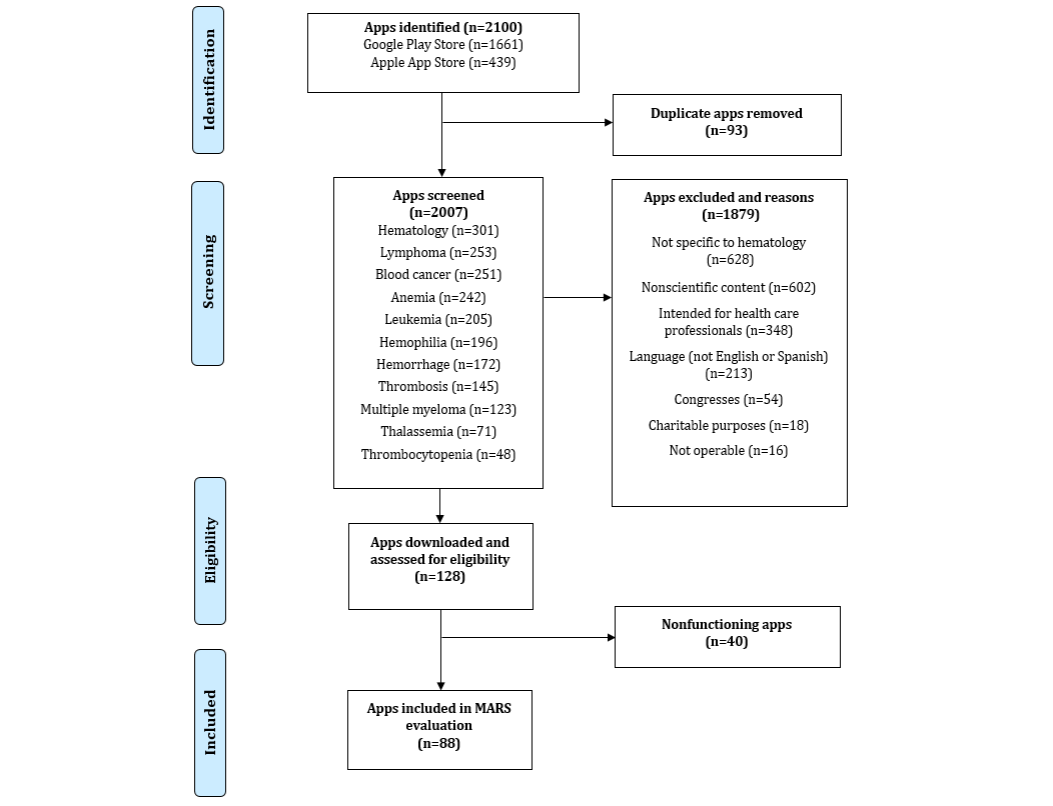
PRISMA (Preferred Reporting Items for Systematic Reviews and Meta-Analyses) flow diagram and app selection. MARS: Mobile App Rating Scale.

A total of 2100 apps were retrieved from the Apple App Store and Android Play Store (1661 Android apps and 439 iOS apps). After screening the description and the screenshots available at the app platforms and deleting apps duplicated, 128 apps were selected as potentially eligible. After downloading and checking the fulfillment of the inclusion criteria, 88 apps were finally included in the descriptive analysis. A flow diagram illustrating the selection and exclusion of apps at various stages of the study is shown in Figure 1.

### Characteristics and Purposes of Included Apps

In total, of the 88 apps, 8 (9%) were found on both digital distribution platforms, whereas 54 (61%) were obtained only from the Android Play Store, and 26 (30%) were only available at the Apple App Store. In addition, of the 88 apps, only 6 (7%) required payment (mean cost: mean €3.16 [US $3.60], SD €1.57 [US $1.79]). Table 1 shows the general characteristics of apps.

**Table 1:** General characteristics of the apps.

Characteristics			Apps, n (%)
**Platform**			
	Android	54 (61)	
	iOS	26 (30)	
	Android and iOS	8 (9)	
**Cost**			
	No	82 (93)	
	Yes	6 (7)	
**Category**			
	Medicine	35 (40)	
	Health and wellness	33 (38)	
	Health and fitness	8 (9)	
	Education	7 (8)	
	Books and reference works	1 (1)	
	Entertainment	1 (1)	
	Lifestyle	1 (1)	
	Simulation	1 (1)	
	Social media	1 (1)	
**Date of the last update**			
	2012	1 (1)	
	2016	2 (2)	
	2017	7 (8)	
	2018	12 (14)	
	2019	19 (22)	
	2020	34 (39)	
	2021	9 (10)	
	Not updated	4 (4)	
**Language**			
	English	80 (92)	
	Spanish	4 (4)	
	English and Spanish	4 (4)	

Regarding purpose, most of the apps were informative (60/88, 68%), followed by preventive (23/88, 26%) and diagnostic (5/88, 6%). Of the 88 apps, a total of 43 apps (49%) were updated in the last year, and 23 apps (26%) were designed and developed with the participation of some kind of health care organization. The distribution of apps regarding hematological conditions was anemia (23/88, 26%), leukemia (12/88, 14%), hemophilia (11/88, 13%), thrombosis (8/88, 9%), thalassemia (7/88, 8%), hematological cancers (leukemia, lymphoma, or myeloma; 5/88, 6%), hemorrhage (5/88, 6%), lymphoma (4/88, 5%), leukemia or lymphoma (3/88, 3%), thrombocytopenia (3/88, 3%), multiple myeloma (2/88, 2%), hematological conditions (2/88, 2%), anemia or hemophilia (1/88, 1%), anemia or thalassemia (1/88, 1%), and hemochromatosis (1/88, 1%). The information on hematological conditions, purpose, app platform, free of cost, updates, developer, and language is shown in Tables 2 and 3.

**Table 2:** Characteristics of the apps analyzed. Apps are presented in alphabetical order, from those that start with "A" to those that start with "I."

Name of the app (developer)	Hematological disease	Purpose				Platform			Free		Updated in the last 12 months		Developed by a health organization		Language	
		I^a^	P^b^	D^c^	iOS		Android							E^d^		S^e^
Alimentos para la anemia (Jotathat)	Anemia	✓					✓	✓		✓						✓
All Blood Disease and Treatment A-Z (Patrikat Softech)	Blood disorders	✓					✓	✓		✓				✓		
ALL Manager (Point of Care)	Leukemia		✓		✓			✓		✓				✓		
ALL Xplained (MedicineX)	Leukemia	✓			✓			✓				✓		✓		
Anemia (Rouseapps)	Anemia	✓					✓	✓		✓				✓		
Anemia (El Makaoui)	Anemia	✓					✓	✓						✓		
Anemia Care Diet & Nutrition (RecoveryBull)	Anemia	✓					✓	✓		✓				✓		
Anemia Home Remedies (StatesApps)	Anemia	✓					✓	✓						✓		
Anemia Home Remedies (Salim Garba Usman)	Anemia	✓					✓	✓						✓		
Bleeder (Hannes Jung)	Hemophilia		✓		✓					✓				✓		
Bleeding After Birth (Jaco Apps)	Hemorrhage	✓					✓	✓		✓				✓		
Bleeding Disorder (Koodalappz)	Hemorrhage	✓					✓	✓		✓				✓		
Blood Cancer (Digital Planete Space)	Hematological cancers	✓					✓	✓						✓		
Blood Cancer Tips (Free Apps For Everyone)	Hematological cancers	✓					✓	✓		✓				✓		
Blood Clot Home & Natural Remedies (Salim Garba Usman)	Thrombosis	✓					✓	✓						✓		
Blood Count Reader free (Yurii Shevchenko)	Anemia			✓			✓	✓						✓		
Blood Diseases (Medico_Guide)	Blood disorders	✓					✓	✓						✓		
Blood Group Genes (Gaurav Mathur)	Anemia or hemophilia			✓	✓			✓		✓				✓		
Caprini DVT Risk (NorthShore University HealthSystem)	Thrombosis	✓			✓			✓				✓		✓		
Childhood Leukemia: A Preventable Disease (FreeCreativity2019)	Leukemia	✓					✓	✓		✓				✓		
CIB—Coagulation Intervention Brigade (LFB Biomedicaments)	Hemorrhage	✓			✓			✓				✓		✓		✓
CLL Manager (Point of Care)	Leukemia		✓		✓			✓		✓				✓		
CLL Watch and Wait Tracker (Lymphoma Canada)	Leukemia or lymphoma		✓		✓		✓	✓		✓		✓		✓		
CML Life (Incyte Corporation)	Leukemia	✓			✓			✓				✓		✓		
CML Today (Leukemia Patient Advocates Foundation)	Leukemia	✓			✓			✓				✓				✓
Diario de INR (Web Factor BV)	Thrombosis		✓		✓					✓				✓		✓
Don’t Walk Alone (Lymphoma Canada)	Leukemia	✓			✓			✓				✓		✓		
Easy Diagnosis—Thalassemia (Sarah Tinmaswala)	Thalassemia	✓					✓	✓		✓				✓		
EasyCoagLite (Loic Letertre)	Thrombosis	✓			✓		✓	✓						✓		
Focus On Lymphoma (Lymphoma Research Foundation)	Lymphoma		✓		✓		✓	✓				✓		✓		
Folate & B12 Counter and Tracker (First Line Medical Communications)	Anemia	✓			✓					✓		✓		✓		
Food For Anemia (MixLabApps)	Anemia	✓					✓	✓		✓				✓		
HaemActive—Fitness for People with haemophilia (NovoNordisk A/S)	Hemophilia	✓					✓	✓		✓		✓		✓		
Haemophilia Pal (Haemophilia Pal)	Hemophilia		✓				✓	✓						✓		
Hemo Control (The Simulation Crew)	Hemophilia		✓		✓			✓		✓				✓		
Hemophilia Disease (Bedieman)	Hemophilia	✓					✓	✓						✓		
Hemophilia Support (MyHealthTeams)	Hemophilia	✓			✓		✓	✓		✓				✓		
Hodgkin Lymphoma Manager (Point of Care)	Lymphoma		✓		✓			✓		✓				✓		
Home Remedies for Anemia (Anil Krishna)	Anemia	✓					✓	✓						✓		
How To Cure Leukemia (Apps How To Apps)	Leukemia	✓					✓	✓						✓		
iClot (Cranworth Medical Ltd)	Thrombosis		✓				✓	✓						✓		
Increase A Low Platelet Count Naturally (FingertipApps)	Thrombocytopenia	✓					✓	✓		✓				✓		
INR Care (Nikhil Patel)	Thrombosis	✓			✓					✓				✓		
Iron Counter and Tracker (First Line Medical Communications)	Anemia		✓		✓					✓		✓		✓		
Iron Deficiency Anemia (Bedieman)	Anemia	✓					✓	✓						✓		
Iron Tracker—Hemochromatosis (IronTracker)	Hemochromatosis		✓				✓	✓				✓		✓		

^a^I: informative.

^b^P: preventive.

^c^D: diagnostic.

^d^E: English.

^e^S: Spanish.

**Table 3:** Characteristics of the apps analyzed. Apps are presented in alphabetical order, from those that start with "J" to those that start with "Z."

Name of the app (developer)	Hematological disease	Purpose				Platform			Free		Updated in the last 12 months		Developed by a health organization		Language	
		I^a^	P^b^	D^c^	iOS		Android							E^d^		S^e^
Juntos contra la anemia (Andres Moran Tello)	Anemia	✓					✓	✓				✓				✓
Leucemia—Sintomas Y Tratamiento—FAQ (Things To Do)	Leukemia	✓					✓	✓						✓		✓
Leukemia: Symptoms And Treatment (The Reyv)	Leukemia	✓					✓	✓		✓				✓		
Leukemia Disease (Bedieman)	Leukemia	✓					✓	✓						✓		
Leukemia Disease Treatment (Woochi Developer)	Leukemia	✓					✓	✓						✓		
LLS CAR T (The Leukemia and Lymphoma Society)	Blood cancers	✓			✓		✓	✓		✓		✓		✓		
LLS Health Manager (The Leukemia and Lymphoma Society)	Blood cancers		✓		✓		✓	✓		✓		✓		✓		
LRF Understanding Lymphoma (Lymphoma Research Foundation)	Lymphoma	✓					✓	✓		✓		✓		✓		
LRFFactSheets (Lymphoma Research Foundation)	Lymphoma	✓			✓			✓		✓		✓		✓		
Microhealth Hemofilia (MicroHealth LLC)	Hemophilia		✓		✓		✓	✓		✓		✓		✓		✓
Mi Hemofilia (Rogelio Robles Tarano)	Hemophilia	✓					✓	✓				✓		✓		
Multiple Myeloma Manager (Point of Care)	Multiple myeloma		✓		✓			✓		✓				✓		
My Blood Count (Sean Bottomley)	Anemia		✓		✓			✓		✓				✓		
My HHT Tracker (Cure HHT)	Hemochromatosis		✓		✓			✓						✓		
My INR (iMonitorMy)	Thrombosis		✓				✓	✓						✓		
My Iron Manager (Good Dog Design Pty Ltd)	Anemia		✓				✓	✓		✓				✓		
Myeloma Cancer Guide (Everyone Learning Apps)	Multiple myeloma	✓					✓	✓		✓				✓		
myPROBE (Design2Code Inc)	Hemophilia		✓		✓			✓		✓				✓		
myWAPPS (Design2Code Inc)	Hemophilia		✓		✓			✓		✓				✓		
NCCN Patient Guides for Cancer (National Comprehensive Cancer Network)	Blood cancers	✓					✓	✓		✓		✓		✓		
PA Pernicious Anaemia (B12 Global Limited)	Anemia		✓		✓									✓		
Pernicious-Anemia Advice (MoreFlow)	Anemia	✓					✓	✓		✓				✓		
Pregnancy & Anaemia (Fumo)	Anemia	✓					✓	✓		✓				✓		
Recetas y consejos para combatir la anemia (App Free Enjoy)	Anemia		✓				✓	✓								✓
Recognize Anemia Disease (Media Clinic)	Anemia	✓					✓	✓						✓		
Recognize Hemophilia Disease (Media Clinic)	Hemophilia	✓					✓	✓						✓		
Recognize Thalassemia Disease (Media Clinic)	Thalassemia	✓					✓	✓						✓		
Recognize Thrombocytopenia (Media Clinic)	Thrombocytopenia	✓					✓	✓						✓		
Sickle Cell Anemia (Fumo)	Anemia	✓					✓	✓		✓				✓		
Sickle Cell Anemia Home remedy (JGWS)	Anemia		✓				✓	✓						✓		
Sickle Cell Disease (Kabirapp)	Anemia	✓					✓	✓						✓		
STB—Stop The Bleed (Uniformed Services University)	Hemorrhage	✓			✓			✓				✓		✓		
SUSOKA (Subrata Saha)	Thalassemia			✓			✓	✓		✓				✓		
Thalassemia Early Detection (ILIANA)	Thalassemia			✓			✓	✓		✓				✓		
Thalassemia Disease (Bedieman)	Thalassemia	✓					✓	✓						✓		
ThaliMe (Curatio Networks Inc)	Thalassemia	✓					✓	✓						✓		
thalTracker (University Health Network)	Thalassemia	✓			✓			✓						✓		
The Cancer App (Interactive Pharma solutions limited)	Blood cancers	✓			✓			✓		✓		✓		✓		
The Seven Types of Anemia (Mrbeli)	Thalassemia or anemia	✓					✓	✓						✓		
Transplant Guidelines (National Marrow Donor Program/Be The Match)	Blood cancers	✓					✓	✓				✓		✓		
Trombocytopenia Disease (Bedieman)	Thrombocytopenia	✓					✓	✓						✓		
VTE Calc (Lindum Medical Ltd)	Thrombosis	✓			✓			✓						✓		

^a^I: informative.

^b^P: preventive.

^c^D: diagnostic.

^d^E: English.

^e^S: Spanish.

### Rating of Apps on the MARS

The specific MARS ratings for each app are shown in Tables 4 and 5. The mean score for the overall quality was 3.03 (SD 1.14), ranging from 1.19 (lowest rated app) to 4.86 (highest rated app). On average, the best-rated section was functionality (mean 3.44, SD 1.07), followed by aesthetics (mean 3.10, SD 1.23), engagement (mean 3.06, SD 1.32), information (mean 2.95, SD 1.09), and app subjective quality (mean 2.61, SD 1.28).

**Table 4:** Mobile App Rating Scale scores of the evaluated apps (rating out of 5). The first half (41/88, 47%) of the apps are presented here.

Name of the app (developer)	Engagement, score	Functionality, score	Aesthetics, score	Information, score	Subjective quality, score	Overall
Multiple Myeloma Manager (Point of Care)	5.00	5.00	5.00	4.57	4.75	4.86
Hodgkin Lymphoma Manager (Point of Care)	5.00	5.00	5.00	4.43	4.75	4.84
Focus On Lymphoma (Lymphoma Research Foundation)	4.90	4.88	4.83	4.43	5.00	4.81
ALL Manager (Point of Care)	5.00	4.88	4.67	4.43	4.88	4.77
CLL Manager (Point of Care)	5.00	4.75	4.67	4.43	4.75	4.72
Transplant Guidelines (National Marrow Donor Program/Be The Match)	4.80	5.00	4.50	4.43	4.50	4.65
HaemActive—Fitness for people with haemophilia (NovoNordisk A/S)	4.90	4.38	5.00	4.57	4.38	4.64
Mi Hemofilia (Rogelio Robles Tarano)	4.30	4.63	4.83	4.79	4.63	4.63
My INR (iMonitorMy)	4.60	4.88	4.17	4.64	4.38	4.53
My Iron Manager (Good Dog Design Pty Ltd)	4.60	4.50	4.50	4.71	4.13	4.49
myWAPPS (Design2Code Inc)	4.90	4.88	4.67	4.07	3.88	4.48
CLL Watch and Wait Tracker (Lymphoma Canada)	4.60	4.00	4.67	4.36	4.75	4.47
Bleeder (Hannes Jung)	4.70	4.50	4.50	4.14	4.50	4.47
Microhealth Hemofilia (MicroHealth LLC)	5.00	4.50	4.17	4.21	4.25	4.43
Iron Tracker—Hemochromatosis (IronTracker)	4.40	4.63	4.67	4.43	4.00	4.42
STB—Stop The Bleed (Uniformed Services University)	4.00	4.63	4.33	4.64	4.50	4.42
PA Pernicious Anaemia (B12 Global Limited)	4.70	4.50	4.50	4.14	4.13	4.39
Hemophilia Pal (Haemophilia Pal)	4.50	4.38	4.17	4.43	4.38	4.37
ThaliMe (Curatio Networks Inc)	4.60	4.50	4.67	3.79	3.88	4.29
My HHT Tracker (Cure HHT)	4.70	4.63	4.50	3.43	4.13	4.28
CML Life (Incyte Corporation)	4.40	4.75	4.67	3.71	3.63	4.23
INR Care (Nikhil Patel)	4.50	4.38	4.67	3.29	4.13	4.19
Diario de INR (Web Factor BV)	4.60	4.13	4.67	3.93	3.63	4.19
NCCN Patient Guides for Cancer (National Comprehensive Cancer Network)	3.10	4.75	4.67	4.36	4.00	4.17
My Blood Count (Sean Bottomley)	4.80	4.38	4.50	3.64	3.50	4.16
LLS Health Manager (The Leukemia and Lymphoma Society)	4.70	4.00	3.83	4.21	4.00	4.15
thalTracker (University Health Network)	4.60	4.50	4.50	3.86	3.25	4.14
Don’t Walk Alone (Lymphoma Canada)	4.90	3.38	4.17	4.21	3.38	4.01
The Cancer App (Interactive Pharma Solutions Limited)	4.30	4.25	4.50	3.71	3.13	3.98
Hemophilia Support (MyHealthTeams)	4.60	3.75	3.67	3.14	4.50	3.93
CML Today (Leukemia Patient Advocates Foundation)	3.70	4.38	3.50	4.29	3.63	3.90
Pernicious Anemia Advice (MoreFlow)	3.10	4.50	4.33	4.00	3.50	3.89
ALL Xplained (MedicineX)	3.10	4.38	4.00	3.71	2.75	3.59
VTE Calc (Lindum Medical Ltd)	3.80	4.38	3.17	3.86	2.63	3.56
myPROBE (Design2Code Inc)	3.80	4.25	4.00	2.71	2.13	3.38
Alimentos para la anemia (Jotathat)	2.70	3.13	3.50	3.43	3.13	3.18
Folate & B12 Counter and Tracker (First Line Medical Communications)	3.10	4.13	2.50	3.36	2.63	3.14
Blood Group Genes (Gaurav Mathur)	4.20	3.25	4.00	2.21	2.00	3.13
Iron Counter and Tracker (First Line Medical Communications)	3.10	4.13	2.50	3.36	2.50	3.12
All Blood Disease and Treatment A-Z (Patrikat Softech)	2.60	3.88	3.00	3.21	2.50	3.04
LRF Understanding Lymphoma (Lymphoma Research Foundation)	3.10	3.38	3.33	3.36	2.00	3.03

**Table 5:** Mobile App Rating Scale scores of the evaluated apps (rating out of 5). The second half (47/88, 53%) of the apps are presented here.

Name of the app (developer)	Engagement, score	Functionality, score	Aesthetics, score	Information, score	Subjective quality, score	Overall
LRFFactSheets (Lymphoma Research Foundation)	3.40	3.38	3.17	3.14	1.88	2.99
Juntos contra la anemia (Andres Moran Tello)	3.00	3.13	3.17	3.07	2.38	2.95
EasyCoagLite (Loic Letertre)	3.30	2.88	3.00	2.79	2.50	2.89
Hemo Control (The Simulation Crew)	3.60	3.00	3.50	2.07	1.88	2.81
Caprini DVT Risk (NorthShore University HealthSystem)	3.70	3.38	2.67	2.57	1.38	2.74
Recognize Thrombocytopenia (Media Clinic)	2.30	3.88	2.00	2.50	1.75	2.49
Thalasemia Early Detection (Iliana)	3.00	3.25	2.67	2.21	1.25	2.48
Recognize Hemophilia Disease (Media Clinic)	2.30	3.88	1.67	2.50	1.88	2.44
Sickle Cell Anemia Home remedy (JGWS)	1.80	2.63	3.00	2.50	2.25	2.44
Recognize Thalassemia Disease (Media Clinic)	2.30	3.88	1.67	2.50	1.75	2.42
Increase A Low Platelet Count Naturally (FingertipApps)	1.80	3.38	2.33	2.43	2.13	2.41
Recognize Anemia Disease (Media Clinic)	2.30	3.25	2.17	2.50	1.75	2.39
Anemia (RouseApps)	2.00	3.38	2.00	2.57	1.88	2.36
LLS CAR T (The Leukemia and Lymphoma Society)	2.40	3.25	2.83	2.07	1.25	2.36
Blood Clot Home & Natural Remedies (Salim Garba Usman)	2.00	3.25	2.67	2.21	1.63	2.35
Anemia Home Remedies (StatesApps)	1.80	3.63	2.17	2.29	1.75	2.33
Bleeding After Birth (JacoApps)	1.90	3.13	2.83	2.14	1.63	2.33
The Seven Types of Anemia (MrBeli)	2.20	3.25	2.00	2.36	1.75	2.31
Sickle Cell Disease (Kabirapp)	1.90	2.38	2.83	2.00	2.25	2.27
Bleeding Disorder (Koodalappz)	2.10	2.50	2.83	2.29	1.63	2.27
Blood Diseases (Medico_Guide)	1.80	2.63	2.27	2.29	2.00	2.18
Childhood Leukemia: A Preventable Disease (FreeCreativity2019)	1.80	2.63	1.83	2.21	2.38	2.17
Trombocytopenia Disease (Bedieman)	1.80	2.75	2.00	2.50	1.75	2.16
Food For Anemia (MixLabApps)	1.80	3.13	2.33	2.14	1.38	2.16
Iron Deficiency Anemia (Bedieman)	1.80	3.00	2.00	2.21	1.75	2.15
Hemophilia Disease (Bedieman)	1.80	2.75	2.00	2.50	1.63	2.14
Thalassemia Disease (Bedieman)	1.70	2.75	2.00	2.50	1.63	2.12
Easy Diagnosis—Thalassemia (Sarah Tinmaswala)	1.90	3.25	1.67	2.00	1.38	2.04
Home Remedies for Anemia (Anil Krishna)	1.80	2.63	2.17	1.93	1.63	2.03
SUSOKA (Subrata Saha)	2.40	2.38	2.17	1.57	1.38	1.98
Leucemia—Sintomas Y Tratamiento—FAQ (Things To Do)	1.60	2.13	2.00	2.43	1.50	1.93
Anemia Care Diet & Nutrition (RecoveryBull)	2.00	2.13	2.00	2.00	1.50	1.93
Sickle Cell Anemia (Fumo)	1.80	2.38	1.83	1.79	1.63	1.88
iClot (Cranworth Medical Ltd)	1.90	2.00	2.17	1.93	1.38	1.87
Pregnancy & Anaemia (Fumo)	1.80	2.38	2.00	1.79	1.38	1.87
Anemia (El Makaoui)	1.50	2.75	2.00	1.64	1.25	1.83
Anemia Home Remedies (Salim Garba Usman)	1.80	2.00	1.83	1.86	1.63	1.82
CIB—Coagulation Intervention Brigade (LFB Biomedicaments)	1.40	1.25	2.50	1.21	1.25	1.52
Leukemia Disease (Bedieman)	1.50	1.50	1.33	1.71	1.13	1.43
Recetas y consejos para combatir la anemia (App Free Enjoy)	1.60	1.50	1.50	1.43	1.00	1.41
Leukemia: Symptoms And Treatment (The Reyv)	1.50	1.75	1.17	1.43	1.13	1.39
Myeloma Cancer Guide (Everyone Learning Apps)	1.20	1.75	1.50	1.50	1.00	1.39
Leukemia Disease Treatment (Woochi Developer)	1.40	1.50	1.17	1.43	1.25	1.35
Blood Cancer (Digital Planete Space)	1.40	1.71	1.17	1.29	1.00	1.31
How To Cure Leukemia (Apps How To Apps)	1.10	1.75	1.17	1.14	1.00	1.23
Blood Cancer tips (Free Apps For Everyone)	1.00	1.63	1.17	1.14	1.00	1.19
Blood-Count Reader free (Yurii Shevchenko)	1.80	1.13	1.00	1.00	1.00	1.19

Comparison by app distribution platform (Apple App Store and Android Play Store) revealed a mean MARS score of 3.85 (SD 0.35) for apps developed for iOS (n=34) and 2.67 (SD 0.30) for apps developed for Android (n=62), resulting in a statistically significant difference (*P*<.001). Apps whose development had been supported by a health organization obtained better scores (mean 3.75, SD 0.29; n=23) than those that had not (mean 2.78, SD 0.31; n=65; *P*<.001). Finally, another statistically significant difference (*P*=.049) was found when the overall MARS scores were analyzed considering whether the apps were free (mean 2.97, SD 0.30; n=82) or required payment (mean 3.92, SD 0.29; n=6; *P*=.049). The comparison by different characteristics is shown in Table 6.

The mean κ coefficient score for the five MARS domains was 0.78. κ values between 0.61 and 0.81 indicate that interrater agreement between the 2 reviewers was substantial. The only item with a score less than 0.61 was ease of use (Table 7).

**Table 6:** Results of the Mobile App Rating Scale evaluation: comparison by different characteristics.

Category	Operating system				Developer				Cost		
	Android (n=62), score	iOS (n=34), score	*P* value	No health organization (n=65), score		Health organization (n=23), score	*P* value	Free (n=82), score		Payment (n=6), score	*P* value
Engagement	2.59	4.16	<.001	2.76		3.88	<.001	2.98		4.12	.04
Functionality	3.09	4.01	<.001	3.23		4.02	.002	3.38		4.29	.04
Aesthetics	2.66	3.91	<.001	2.82		3.90	.002	3.04		3.89	.10
Information	2.64	3.52	<.001	2.70		3.67	<.001	2.90		3.70	.08
Subjective quality	2.26	3.34	<.001	2.37		3.29	.002	2.54		3.58	.052
Overall	2.67	3.85	<.001	2.78		3.75	<.001	2.97		3.92	.049

**Table 7:** Kappa score and interrater reliability for the Mobile App Rating Scale domains.

Domain		Weighted Cohen κ	Agreement, %
**Engagement**		0.82	93.1
	Entertainment	0.63	86.7
	Interest	0.72	90.4
	Customization	0.90	95.2
	Interactivity	0.84	92.6
	Target group	0.73	90.4
**Functionality**		0.69	90.6
	Performance	0.67	88.5
	Ease of use	0.54	87.1
	Navigation	0.64	87.9
	Gestural design	0.71	90.3
**Aesthetics**		0.80	93.1
	Layout	0.76	91.7
	Graphics	0.76	90.7
	Visual appeal	0.78	92.4
**Information**		0.80	93.5
	Accuracy of the app in the description (Apple App Store and Android Play Store)	0.77	93.1
	Goals	0.78	92.8
	Quality of information	0.73	91.0
	Quantity of information	0.67	88.6
	Visual information	0.63	86.4
	Evidence base	0.91	96.3
	Credibility	0.84	94.3
**Subjective quality**		0.80	92.8
	Would you recommend this app to people who might benefit from it?	0.78	91.5
	Would you pay for this app?	0.86	94.2
	How many times do you think you would use this app in the next 12 months if it was relevant to you?	0.77	90.3
	What is your overall star rating of the app?	0.79	92.2

## Discussion

### Principal Findings

This is the first study to provide a systematic search and ranking of apps for patients diagnosed with hematological conditions available in the Apple App Store and Android Play Store, using the MARS as a standardized methodology for the classification, assessment, and validation of these apps.

We found that there were more apps available in the Android Play Store than in the Apple App Store, as mentioned in other studies [8,11,24], which can imply that uploading an app into the Android Play Store is an easier process. We observed that almost half of the apps (43/88, 49%) had been updated in the last year, as previously reported [25]. Considering hematology as a medical field that is constantly growing in complexity and extending its therapeutic arsenal, this low rate of app content actualization is insufficient [26].

Of 88 apps, only 23 (26%) were designed with the participation of some kind of health organization. The absence of health care professionals in the development of health apps continues to be raised time and time again. Amor-García et al [11] observed that only 15.2% of apps for patients with genitourinary cancer involved health professionals in their design process. When reviewing apps for medication management, Tabi et al [27] observed a similar result (14.6%). It would seem crucial that health care professionals be involved in the creation of medical apps; however, this scarcely happens. Moreover, the fact that most health-related apps are free favors accessibility [27].

Our results expose the high prevalence of informative apps (60/88, 68%), as reported by other authors [6,11]. The majority of these apps provide generic data about one or several pathologies, including symptoms, diagnostics, and treatment, focusing solely on education. One-third of the total of informative apps is intended for patients with anemia, which highlights the interest in anemia self-management, as it is the most common blood disorder globally [18]. Preventive apps are less numerous (23/88, 26%), although their quality and performance are significantly higher. These apps focus on handling the pathology after diagnosis, allowing for treatment and laboratory values tracking and recording of symptoms and adverse events. We found these types of apps the most appropriate and useful for patients with hematological conditions because many blood conditions require chronic and complex pharmacologic treatment [28,29]. Only 5 diagnostic apps were evaluated. It is worth mentioning *STB—Stop The Bleed*, an app designed to help anyone learn how to safely and effectively deal with life-threatening bleeding, which has demonstrated the potential of mobile apps in emergency scenarios [30]. The other 4 diagnostic apps are screening tools based on hematological parameters, questionnaires, and gene traits. Its objectives are to predict blood groups or certain hereditary pathologies, such as hemophilia or thalassemia. The main limitation is again the lack of evidence-based content, which in this case could mislead patients into not seeking professional advice. The potential of apps to be implemented as remote diagnostic tools for hematological conditions is very high. This is the case of AnemoCheck Mobile, an app that estimates hemoglobin levels by analyzing the color of fingernail beds and detects anemia, serving as a completely noninvasive anemia screening tool [31].

The MARS has demonstrated its potential as a simple, reliable, and flexible health care app-quality rating scale [21]. It analyzes the quality of an app by evaluating 23 items, grouped into 5 domains, and rating on a 5-point scale. Our study showed a mean score of 3.03, considering a score of 3 as *acceptable*. This result is similar to the scores showed by other authors using the MARS to evaluate health apps for other conditions. The mean score found by Salazar et al [5] for apps designed for chronic pain management was 3.17, and Kwan et al [6] showed a mean score of 3.48 for apps targeted at patients with spondyloarthritis, out of 18 and 5 apps evaluated, respectively. Knitza et al [24] reviewed 28 rheumatology apps and obtained an overall MARS score of 3.85. The median overall MARS score of the analysis of 34 apps targeted toward supporting heart failure symptom monitoring was 3.4 [32]. In a larger sample study, Amor-García et al [11] evaluated 46 apps for patients with genitourinary cancers and found a mean score of 2.98. It is worth noting that our study encompasses a higher number of apps evaluated than any of the studies cited. Thus, the overall quality of health apps in digital platforms is moderate, and there remains considerable scope for improvement. Of the 88 apps, 41 (47%) hematological apps obtained a score of at least 3 points, meaning that more than half of the apps for hematological conditions do not meet acceptable criteria for quality and content. Moreover, of the 88 apps, only 28 (32%) exceeded 4 points in the overall score.

MARS ratings ranged from 1.19 (*Blood Count Reader*) to 4.86 (*Multiple Myeloma Manager*), indicating the highly inconsistent quality of apps. The apps with the highest scored were *Multiple Myeloma Manager*, *Hodgkin Lymphoma Manager*, *Focus On Lymphoma*, *ALL Manager*, and *CLL Manager*. All of them were exclusive to the Apple App Store, except *Focus On Lymphoma*, which was available in both platforms. These apps showed high scores in the engagement and functionality domains. The main characteristic that defines these top-rated apps was the active patient participation, offering wide treatment and symptom monitoring options, reminders, and schedules edition. The five apps with the highest score had a plain preventive purpose, whereas informative apps scored lower on the MARS despite being more frequent.

The comparison by operating system showed a statistically significant difference favoring iOS apps over Android apps in all 5 MARS domains, a tendency that has been observed in a similar evaluation about genitourinary apps [11]. The reason could be that the Apple App Store has stricter standards to include apps.

Although we observed that only 26% (23/88) of the apps involved the participation of health professionals in their design, their quality was significantly higher. The lack of health professional involvement is a constant that has already been highlighted by several authors, expressing their concern about app content and compromising patient safety [33-35]. However, 4 of the best apps (*Multiple Myeloma Manager*, *Hodgkin Lymphoma Manager*, *ALL Manager*, and *CLL Manager*) were developed by @Point of Care, a platform consisting of nonmedical stakeholders and dedicated to creating medical apps for patients and clinicians. @Point of Care has designed apps focused on diverse pathologies, some of them obtaining considerably high MARS scores in other studies similar to ours [11]. The analysis by cost revealed another statistically significant difference, positioning payment apps ahead of free apps in terms of quality, although the fact that only 6 hematological apps were not free and all of them were developed for iOS can destabilize the comparison.

Functionality was the domain that scored the highest on the MARS test, as described by other authors [11,36]. This implies that the apps are easy to navigate and efficient. Leaving subjective quality aside, engagement and information were the domains with the lowest MARS scores. Engagement reflects the capacity of the app to be personalized by the user. Patients usually search for a health app that allows for medication management, clinical and analytical parameter register, and symptom tracking [28,37]. Patients with hematological conditions would benefit significantly from this type of assistance, as several blood conditions demand constant patient monitoring and high adherence to treatment for a better health outcome [18,30]. *My INR*, *INR Care*, and *Diario de INR* are apps that allow anticoagulated patients to record and track their international normalized ratio readings and antivitamin K dosages. They could help improve adherence and avoid potential complications, such as the risk of bleeding or clots. *HaemActive* is a fitness app especially tailored to patients with hemophilia, who require special exercises that imply a minimal risk of bleeding. The app includes weekly training planning, explainer videos, and easy customization. In addition, patients expressed their interest in using health apps to communicate with their physicians [28,38]. Concerning the information domain, there is 1 specific item assessing the *evidence base*, which explores the extent to which the app has been scientifically tested. However, this item was excluded from all calculations, as no clinical studies to support the effectiveness and safety of any of the apps could be found. Thus, empirical studies should be conducted for apps to determine their clinical impact on outcomes for patients diagnosed with hematological conditions [13].

### Recommendations for Health App Development

The number of health apps available and studies reviewing their quality is steadily growing, which will help health professionals to recommend apps to patients. This activity acquires even further relevance, considering the still little control from regulatory authorities over health app development. We have observed that the main issues that need to be addressed when designing health apps are as follows: no participation of health organizations in app development, questionable sources of information, and deficient interactivity and personalization options [35]. Production of medical apps from nonmedical stakeholders has benefits in terms of creativity in the design of apps. However, it must be combined with clinician assistance to boost the credibility of medical information with such apps. Concerning patients with hematological conditions, registering analytical information, treatment prescribed, and symptoms is highly recommendable for apps to help them in their care.

### Limitations

First, only apps available in the Android Play Store and Apple App Store, with contents in English or Spanish and accessed from a Spanish IP address were included, assuming the possibility of having missed some other apps dedicated to hematological conditions. Another limitation could be that app quality was assessed using the MARS, which is limited by the subjectivity of the evaluators. Nevertheless, this issue is partially addressed by the high interrater reliability of the data analyzed by the 2 independent researchers. We believe that this evaluation should allow health care professionals and patients to identify which apps meet minimum standards of quality and safety in their content.

### Conclusions

We provide the first systematic review of apps related to hematological conditions, identifying 88 apps and rating them using the MARS. The study shows great heterogeneity among their quality. Many of these apps emerge as tools for consulting information, being the most frequent functionality, although not the highest rated. A very small number of them offer a comprehensive self-management approach incorporating evidence-based strategies. Only 26% (23/88) of the apps were developed with the assistance of health care professionals. The top 5 rated apps—*Multiple Myeloma Manager*, *Hodgkin Lymphoma Manager*, *Focus On Lymphoma*, *ALL Manager*, and *CLL Manager*—allowed for active patient participation and app personalization. Higher scores in quality were observed in iOS apps, apps developed by health organizations, and payment apps.

## Abbreviations

MARS: Mobile App Rating Scale
PRISMA-P: Preferred Reporting Items for Systematic Review and Meta-Analysis Protocols
